# Endobronchial ultrasound-guided transbronchial needle aspiration in the diagnosis of intrathoracic lymph node metastases from extrathoracic malignancies

**DOI:** 10.1007/s10585-012-9556-3

**Published:** 2012-11-30

**Authors:** Jose Sanz-Santos, Beatriz Cirauqui, Estefania Sanchez, Felipe Andreo, Pere Serra, Eduard Monso, Eva Castellà, Mariona Llatjós, Miguel Mesa, Juan Ruiz-Manzano, Rafael Rosell

**Affiliations:** 1 Pulmonology Department, Hospital Germans Trias i Pujol, Badalona, Spain; 2 Catalan Institute of Oncology, Badalona, Spain; 3 Departament de Medicina, Universitat Autònoma de Barcelona, Bellaterra, Spain; 4 Ciber de enfermedades respiratorias CiBeRes, Bunyola, Spain; 5 Pulmonology Department, Hospital Parc Taulí, Sabadell, Spain; 6 Pathology Department, Hospital Germans Trias i Pujol, Badalona, Spain; 7 Thoracic Surgery Department, Hospital Germans Trias i Pujol, Badalona, Spain; 8 Dexeus University Institute, Barcelona, Spain; 9 Bronchoscopy Unit, Pulmonology Department, Hospital Germans Trias i Pujol, Carretera de Canyet, s/n, 08916 Badalona, Spain

**Keywords:** Endobronchial ultrasound, Transbronchial needle aspiration, EBUS-TBNA, Mediastinal lymph node metastases, Extrathoracic malignancy, Immunohistochemistry

## Abstract

Intrathoracic lymph node enlargement is a common finding in patients with extrathoracic malignancies. Endobronchial ultrasound-guided transbronchial needle aspiration (EBUS-TBNA) is a technique that is commonly used for lung cancer diagnosis and staging but that has not been widely investigated for the diagnosis of enlarged mediastinal and lobar lymph nodes in patients with extrathoracic malignancies. We conducted a retrospective study of 117 patients with extrathoracic malignancies who underwent EBUS-TBNA for diagnosis of intrathoracic lymph node enlargement from October 2005 to December 2009 and compared the EBUS-TBNA findings with the final diagnoses. EBUS-TBNA diagnosed mediastinal metastases in 51 of the 117 (43.6 %) cases and gave an alternate diagnosis or ruled out the presence of malignancy in 35 (56.4 %). Fourteen of these 35 patients underwent further surgical investigation, while the remaining 21 had clinical and radiological follow-up for 18 months. No false negatives were found in the surgery group. In the follow-up group, 13 patients had stable or regressive lymphadenopathy, and eight developed clinicoradiological progression and were assumed to have been false negatives by EBUS-TBNA. The sensitivity and negative predictive value of EBUS-TBNA were 86.4 and 75 %, respectively. Immunohistochemical staining (IHC) was performed in 80.4 % of the samples obtained by EBUS-TBNA. In samples obtained from ten patients with metastatic breast cancer, estrogen receptor expression was successfully assessed in eight patients and progesterone receptor and human epidermal growth factor receptor 2 in four. EBUS-TBNA is an accurate procedure for the diagnosis of thoracic lymph node metastases in patients with extrathoracic malignancies and should be an initial diagnostic tool in these patients. Furthermore, EBUS-TBNA can obtain high-quality specimens from metastatic lymph nodes for use in molecular analyses.

## Introduction

Intrathoracic hilar and/or mediastinal nodal enlargement in patients with concurrent or previously diagnosed extrathoracic malignancies is a common finding both by computed tomography (CT) or positron emission tomography-computed tomography (PET-CT). Nodal enlargement can be found at the time of the initial diagnosis, which may affect staging and therefore be crucial for the choice of treatment, or it may be identified during or after the course of a radical treatment, which may imply a disease relapse. In addition, nodal enlargement may be from a primary intrathoracic malignancy, such as lung cancer, which will require a change in treatment. In all these situations, intrathoracic nodal enlargement requires pathologic confirmation and generally represents a challenge for the clinician.

For many years, mediastinoscopy was the only diagnostic procedure for mediastinal lymphadenopathy. However, this surgical technique requires general anesthesia and is not the most suitable for patients undergoing chemotherapy. Over the last years, endoscopy ultrasound-guided fine needle aspiration (EUS-FNA) has proven to be an alternative to surgery for sampling mediastinal nodes in these patients [1, 2], but it cannot reach all the nodal stations.

Endobronchial ultrasound-guided transbronchial needle aspiration (EBUS-TBNA) is a relatively novel technique whose usefulness in the diagnosis and staging of lung cancer [3] is widely recognized. However, few studies have focused on the use of EBUS-TBNA in the diagnosis of intrathoracic lymph node metastases in patients with extrathoracic malignancies [4–7]. Furthermore, although EBUS-TBNA has been used successfully to collect samples for molecular analyses in lung cancer patients [8], only one single study has reported the feasibility of EBUS-TBNA to obtain samples from metastatic extrathoracic malignancies that allow the performance of molecular analyses [9].

We have retrospectively assessed the value of EBUS-TBNA for the diagnosis of mediastinal lymph node metastases in patients with extrathoracic malignancies. In addition, we have examined the feasibility of obtaining sufficient high-quality tissue samples with EBUS-TBNA for ancillary molecular analyses which can provide additional diagnostic information for these patients.

## Patients and methods

### Patients

We retrospectively analyzed the clinical files of all patients with a concurrent or a previously diagnosed extrathoracic malignancy who were referred to our institution for EBUS-TBNA because of suspected intrathoracic nodal metastases from October 2005 to December 2009. The clinical suspicion of metastases was based on nodal enlargement (short axis > 10 mm) on CT (with or without lung lesions) [10] and/or on 2-fluoro-2-deoxy-d-glucose (FDG) uptake on PET-CT in all cases. Patients with a previous or concurrent pathologic diagnosis of intrathoracic malignancy previously to the performance of EBUS-TBNA were not included in the study.

### Procedures

EBUS-TBNA was performed in an outpatient setting using a flexible bronchoscope (BF-UC160F-OL8, Olympus Optical Co Ltd., Tokyo, Japan) with a distal probe capable of producing linear parallel scans of the mediastinal and peribronchial tissues and a working channel suited to the performance of TBNA under direct ultrasound guidance. Local anesthesia and conscious sedation were achieved using topical lidocaine spray and intravenous midazolam, respectively [11]. Mediastinal and lobar nodes with a short-axis diameter of ≥5 mm identified during the procedure were sampled under direct ultrasound visualization with a 22-gauge cytology needle specially designed for EBUS-TBNA (NA-201SX-4022, Olympus Optical Co Ltd.).

The aspirates were recovered and placed on slides, fixed with 95 % ethanol and stained with haematoxylin for rapid on-site evaluation by a cytopathologist. An immediate assessment was given after each pass. Nodes were classified as “normal tissue negative for malignancy” when the sample contained 40 lymphocytes per high-power field in cellular areas of the smear and/or clusters of pigmented macrophages and contained no neoplastic cells or as “metastatic” when recognizable groups of malignant cells were present [12]. After this immediate assessment, Papanicolaou staining was completed in the laboratory. Whenever the cytopathologist considered it to be necessary, additional material was obtained and processed as cell blocks for ancillary studies. Cell blocks were prepared by air-drying the slides to clot and scraping them into 10 % formalin for subsequent processing in the laboratory. Blocks were embedded in paraffin and sectioned (5 μm thickness). Routine haematoxylin–eosin staining was used on cell-block sections, and IHC was used whenever this was needed for tumor origin identification.

In patients with metastatic breast cancer the expression of estrogen receptor (ER), progesterone receptor (pgR) and human epidermal growth factor receptor 2 (HER2) was analyzed in EBUS-TBNA recovered material. ER and PgR were evaluated using IHC with the anti-estrogen receptor antibody 6F11 and the anti-progesterone receptor antibody 5D10 respectively (Novocastra, Newcastle Upon Tyne, England). The threshold values for reporting positivity were 1 % of tumor cells [13]. For HER2, staining was performed using Herceptest (DakoCytomation, Carpinteria, CA, USA), and positive cases were confirmed by means of fluorescence in situ hybridization (FISH; Vysis PathVysion, Downers Grove, IL, USA).

When EBUS-TBNA findings were positive for malignancy, they were assumed to be true positives and no further tissue confirmation was requested. Patients in whom EBUS-TBNA did not unequivocally show the presence of malignancy or an alternate benign diagnosis were referred for additional investigations including mediastinoscopy or thoracotomy to obtain a reference pathology result. Clinical and radiological follow-up for at least 18 months was used if the clinician judged this was sufficient. No patients were lost to follow-up.

### Statistical analysis

Data were analyzed using SPSS software version 17.0 (SPSS Inc., Chicago, IL, USA). Results were expressed as absolute and relative frequencies for categorical variables and as means and standard deviations (SD) or as medians and interquartile ranges (IQR), for continuous variables. Specificity and positive predictive value were assumed to be 100 %. Sensitivity (number of true positives/number of true positives + number of false negatives), negative predictive value (NPV; number of true negatives/number of true negatives + number of false negatives) and accuracy (number of true positives + number of true negatives/number of true positives + number of false positives + number of true negatives + number of false negatives) were calculated for the diagnosis of intrathoracic nodal metastases from extrathoracic malignancies.

## Results

**Fig. 1:**
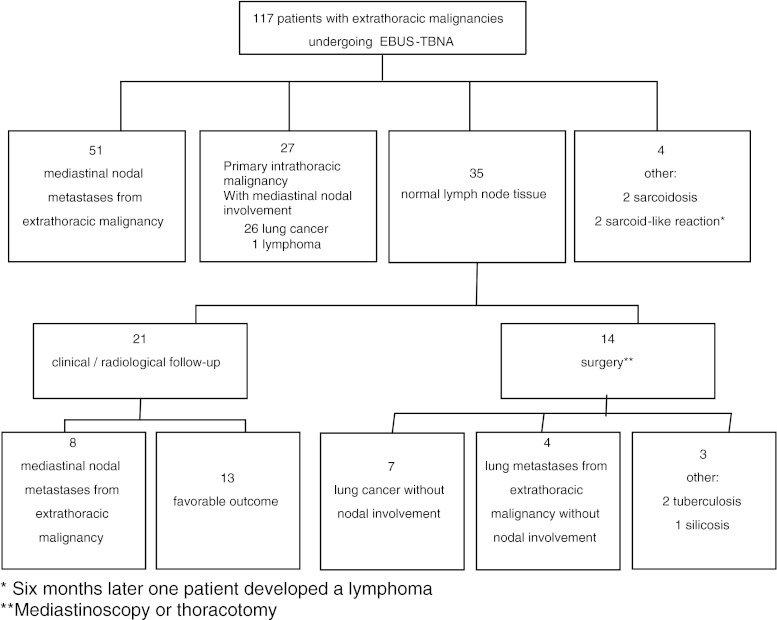
Distribution of patients by EBUS-TBNA findings, follow-up and final diagnosis

One hundred and seventeen patients were reviewed. Table 1 shows the patient characteristics. Head and neck, colorectal, breast and prostate carcinoma represented 60 % of the malignancies included. Figure 1 shows the disposition of patients by EBUS-TBNA findings and follow-up.

**Table 1:** Patient characteristics (*N* = 117)

Characteristic	*N* (%)
Age (years), mean(SD)	65.3 (12.3)
Gender (male), *n* (%)	77 (66)
Extrathoracic malignancy *n* (%)	
Head and neck carcinoma	21 (18)
Colorectal carcinoma	19 (16.4)
Breast carcinoma	18 (15.4)
Prostate carcinoma	12 (10.2)
Urothelial carcinoma	9 (7.7)
Renal carcinoma	7 (6)
Bladder carcinoma	5 (4.3)
Sarcoma	5 (4.3)
Stomach carcinoma	4 (3.5)
Unknown origin carcinoma	4 (3.4)
Melanoma	3 (2.5)
Endometrial carcinoma	2 (1.7)
Thyroid carcinoma	2 (1.7)
Other*	6 (4.8)
Extrathoracic malignancy status	
Previously diagnosed	65 (55.5)
Concurrent	52 (44.1)
During diagnosis	16 (13.7)
During treatment	36 (30.8)
CT findings	
Intrathoracic nodal enlargement without lung lesion	46 (39.3)
Intrathoracic nodal enlargement with solitary nodule/mass	44 (37.6)
Intrathoracic nodal enlargement with multiple nodules/masses	27 (23.1)

* cervical, ovarian, pancreas, adrenal, testis, extrathoracic lymphoma

EBUS-TBNA identified intrathoracic nodal metastases from an extrathoracic malignancy in 51 (43.5 %) cases, a primary intrathoracic malignancy with lymph node involvement in 27 (23 %) cases (26 lung cancer, 1 lymphoma), and an alternative benign diagnosis in four (3.4 %) cases (2 sarcoidosis, 2 sarcoid-like reaction—non-caseating granulomatous inflammation, clinically inconsistent with sarcoidosis, with fungal and mycobacterial negative cultures) (Fig. 1). For all these patients, no histological confirmation was requested. In the two patients with sarcoid-like reaction, clinical and radiological follow-up was carried out to confirm or rule out a possible tumor as the cause [14]. After 6 months, one of these two patients developed a lymphoma.

EBUS-TBNA found normal lymph node tissue in 35 (29.9 %) patients, 14 (11.9 %) of whom underwent surgery to obtain a reference pathology result. Seven (6 %) of these patients had lung cancer without nodal involvement, four (3.4 %) had a lung metastasis from an extrathoracic malignancy without nodal involvement, and in the remaining three patients an alternative benign diagnosis was established (one case of silicosis and two cases of nodal tuberculosis). These three cases that have a diagnosis of normal lymph node tissue established with EBUS-TBNA but an alternative benign disease after surgery, although considered as true negatives for malignancy, were not included in the analysis of NPV. No false negatives were found among the 14 patients who underwent surgery (Fig. 1).

The remaining 21 (17.9 %) patients with neither an unequivocal diagnosis of metastases nor an alternate diagnosis underwent clinical and radiological follow-up for at least 18 months. In 13 (11.1 %) of these patients, stable or regressive lymphadenopathy, consistent with a benign diagnosis, was confirmed. In eight (6.8 %) of these patients, radiological follow-up found mediastinal and/or pulmonary progression, and the EBUS-TBNA findings were categorized as false negatives (Fig. 1). Most of these patients had thoracic progression, including nodal enlargement and/or multiple pulmonary nodes or masses, or extrathoracic metastases, including abdominal or intracranial metastases, and due to ethical reasons or because the patients rejected surgery, no histological confirmation was requested. Table 2 shows the final diagnoses for all 117 patients.

**Table 2:** Final diagnosis of 117 patients with extrathoracic malignancy undergoing EBUS-TBNA for diagnosis of intrathoracic lymph node enlargement, *n* (%)

Mediastinal nodal metastases from extrathoracic malignancy	59 (50.4)
Pulmonary metastases from extrathoracic malignancy without nodal involvement	4 (3.4)
Intrathoracic malignancy	
Lung cancer	33 (28.2)
Lymphoma	1 (0.8)
Benign lymph node disease	
Normal lymph node tissue	13 (11.1)
Sarcoidosis	2 (1.7)
Tuberculosis	2 (1.7)
Sarcoid-like reaction	2 (1.7)
Silicosis	1 (0.8)

The sensitivity, negative predictive value (NPV) and accuracy of EBUS-TBNA for the diagnosis of nodal metastases from extrathoracic malignancies were 86.4 % (51/51 + 8), 75 % (24/24 + 8) and 90.3 % (51 + 24/51 + 0 + 24 + 8) respectively. No complications appeared during the EBUS-TBNA procedure or in the 2 weeks following the procedure.

Among the 51 patients in whom EBUS-TBNA found lymph node metastases (Table 3), colorectal and breast carcinoma were the most frequent primary malignancies (10 [19.6 %] patients each). Thirty-one (60.7 %) patients showed nodal enlargement on EBUS, and 91 % of the patients having a PET-CT showed high FDG avidity. Half of the patients had extrathoracic spread. Twenty-nine patients (56.8 %) had a median disease-free status of 58 months (IQR, 45–112) from diagnosis of the primary malignancy. Five had had a previous pulmonary metastasectomy, with a mean time of 29 months from surgery. Eleven (21.6 %) patients presented with a single mass or nodule on CT scans and could thus have been candidates for metastasectomy, but surgery was ruled out based on the EBUS-TBNA findings.

**Table 3:** Characteristics of patients with intrathoracic nodal metastases from extrathoracic malignancies diagnosed by EBUS-TBNA (*N* = 51)

Characteristic	*N* (%)
Age (years), m (SD)	62.2 (13.2)
Gender (males)	27 (53)
Intra/extrathoracic spread	24/27
Malignancy	
Colorectal carcinoma	10
Breast carcinoma	10
Unknown origin carcinoma	4
Renal carcinoma	4
Stomach carcinoma	3
Head and neck carcinoma	3
Prostate carcinoma	2
Thyroid carcinoma	2
Urothelial carcinoma	2
Melanoma	2
Sarcoma	2
Others	7
Extrathoracic malignancy status	
Previously diagnosed	29 (56.9)
Concurrent	22 (43.1)
During diagnosis	17 (33.3)
During treatment	5 (9.8)
Location of malignant nodes	
Lobar	11 (21.6)
Mediastinal	40 (78.4)
Upper right paratracheal	1 (2)
Subcarinal	19 (37.3)
Lower left paratracheal	3 (5.9)
Lower right paratracheal	17 (33.3)
Characteristics of malignant nodes	
FDG avidity**	9.5 ± 4.6
Size (short-axis diameter)***	11.9 (8.4−8)

* one case each of bladder, cervical, endometrial, testis, ovarian, pancreatic and adrenal cancer

** In Standardized Uptake Value (SUV) (SD)

*** In mm (range)

**Fig. 2:**
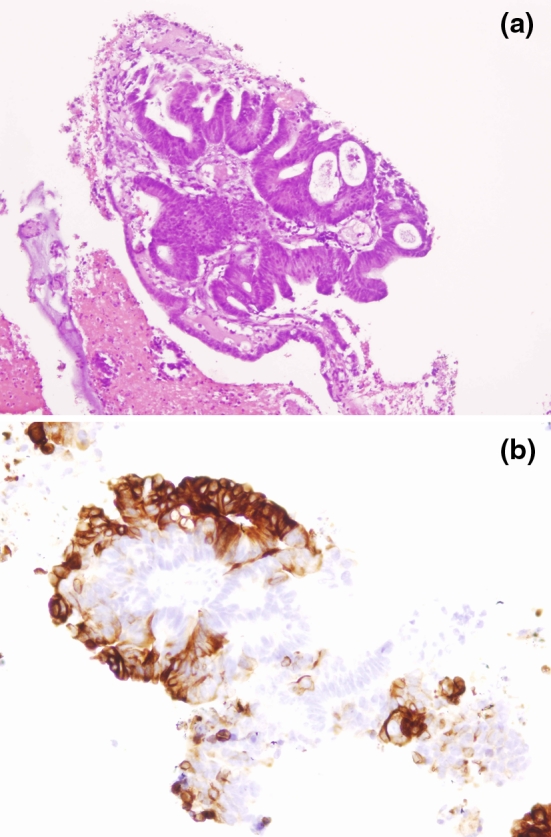
**a** Cell block (haematoxylin–eosin 20×): mediastinal lymph node metastasis from colon adenocarcinoma. **b** At the immunohistochemical analysis, tumor cells were positive for keratin 20 (20×). Immunohistochemistry was consistent with metastasis from colon cancer

**Fig. 3:**
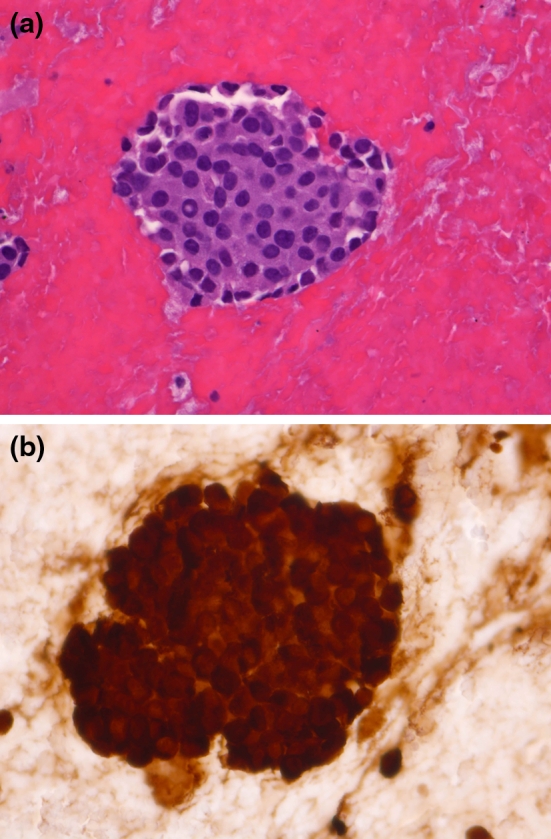
**a** Cell block (haematoxylin–eosin 40×): mediastinal lymph node metastasis from medullary thyroid carcinoma. **b** At the immunohistochemical analysis, tumor cells were positive for calcitonin (40×). Immunohistochemistry was consistent with metastasis from medullary thryroid carcinoma

Cell blocks were obtained from 47 (92 %) of the 51 patients with lymph node metastases diagnosed by EBUS, and IHC was performed in 41 (80.5 %) (Figs. 2, 3). IHC was not considered necessary for the diagnosis in three (5.8 %) patients who had had pharyngeal or laryngeal squamous cell carcinoma and seven (13.7 %) additional patients with different malignancies since the recovered material was morphologically consistent with the previously diagnosed extrathoracic malignancy.

In the ten patients with metastatic breast carcinoma, ER expression was assessed in eight cases and PgR and HER2 in four. PgR status had changed in only one patient, while ER and HER2 status showed no change.

## Discussion

Our study confirms that EBUS-TBNA can diagnose thoracic lymph node metastases from extrathoracic malignancies with sensitivity and NPV similar to those of the two previous major studies [4, 5] and to findings in lung cancer staging [3]. Moreover, in our cohort of 117 patients, implementation of EBUS-TBNA obviated invasive surgical diagnostic procedures in nearly three quarters of the patients and allowed IHC and additional molecular analysis to be performed in more than 80 % of the cases.

In recent years, the development of novel therapies in cancer has led to an improvement in overall survival time [15]. Cancer patients are now given periodic medical controls, and the detection of thoracic lymphadenopathy in patients with extrathoracic malignancies is no longer uncommon [16]. This progress has been accompanied by a parallel advance in minimally invasive techniques, such as EUS-FNA and EBUS-TBNA, which have proven to be a viable alternative to mediastinoscopy. However, mediastinoscopy requires general anesthesia, increasing both costs and patient risk [17, 18]. Several studies [1, 2] have evaluated the use EUS-FNA as a substitute for mediastinoscopy. In a retrospective series of 75 patients, EUS-FNA had a sensitivity of 86 % and NPV of 72 % [1], and in a short prospective study of 20 patients, the sensitivity of EUS-FNA was 68 % [2]. However, compared to EUS-FNA, EBUS-TBNA has the advantage of its bilateral hilar and mediastinal reach while EUS-FNA reaches only the left paratracheal, aorto-pulmonary window, subcarinal and paraesophageal mediastinal lymph node stations. In our series, ten (21.6 %) hilar and 18 (35.3 %) right paratracheal metastatic nodes would have been inaccessible by EUS-FNA.

EBUS-TBNA has long been used for diagnosing and staging lung cancer, but now there is a wealth of evidence to suggest that it has other uses [19]. Several studies have focused on the use of EBUS-TBNA for the diagnosis of sarcoidosis [20], but few have used it specifically for the detection of thoracic nodal metastases from extrathoracic malignancies [4–7]. In a retrospective series of 92 patients, EBUS-TBNA had a sensitivity of 85 % and a NPV of 76 % [5], and a similar study of 161 patients reported a sensitivity of 87 % and a NPV of 73 % [4]. In the present study, sensitivity and NPV were 86.4 and 75 %, respectively, which is similar to these two previous studies [4, 5]. The NPV of 75 % reported in this and in previous studies, however, justifies the referral of patients with neither an unequivocal diagnosis of malignancy nor an alternative diagnosis for additional tests, which may include mediastinoscopy.

Of the 44 patients with a single node mass or nodule on CT scans who were candidates for surgery, intrathoracic lymph node metastases from an extrathoracic malignancy were detected by EBUS in 11 (25 %), and surgery was ruled out for these patients based on the EBUS-TBNA findings. As the likelihood of a curative treatment after a metastasectomy depends on lymph node involvement, the European Society of Thoracic Surgeons recommends excluding patients with lymph node metastases from pulmonary metastasectomy with intent to cure. The incidence of lymph node involvement in patients undergoing pulmonary metastasectomy is estimated to be around 20 % [21]. The use of mediastinoscopy for selecting patients for surgery is unusual [22], and only one study [23] has examined the usefulness of mediastinoscopy in patients with lung metastases eligible for surgery; lymph node metastases were found in 10 % of the patients. In this clinical setting, EBUS-TBNA is thus a preferable approach, and the present study demonstrates the capabilities of EBUS-TBNA for the accurate selection of patients for therapeutic metastasectomy, as had been suggested by preliminary studies [24].

The present study has confirmed the ability of EBUS-TBNA to obtain high-quality specimens for ancillary studies, such as IHC and molecular analyses. In fact, IHC was performed in 80.4 % of the 51 patients diagnosed with mediastinal lymph node metastases. Molecular analysis is commonly used in samples obtained by EBUS-TBNA from patients with lung cancer, where they have been used to distinguish different types of non-small-cell lung cancer [25, 26] and to detect epidermal growth factor mutations [27] and the EML4-ALK fusion gene [8]. In the present study, among ten patients with metastatic breast cancer, it was possible to analyze ER expression in eight patients and PgR and HER2 expression in four. Since ER, PgR and HER2 expression levels can change in metastatic lesions in patients with breast cancer [28] and lead to drug resistance, a biopsy of suspected metastatic lesions is recommended in these patients for the analysis of ER, PgR and HER2 expression [29, 30]. The present study confirms that EBUS-TBNA is a feasible minimally invasive method for obtaining samples for these molecular analyses.

There are a number of limitations in this study. Firstly, the fact that it is a retrospective study implies a certain selection bias. Secondly, no histological confirmation was obtained for cases diagnosed as negative by EBUS-TBNA, including those cases identified as false negatives during clinical and radiological follow-up. A prospective study with well-defined criteria for inclusion and histological confirmation could overcome both these limitations.

In conclusion, EBUS-TBNA is a simple, safe and accurate procedure for the diagnosis of thoracic lymph node metastases in patients with a concurrent or previously diagnosed extrathoracic malignancy. Our findings lead us to recommend the use of EBUS-TBNA as an initial diagnostic technique in these patients. Furthermore, EBUS-TBNA can obtain high quality specimens from metastatic lymph nodes for use in molecular analyses.
